# Reply to the letter to the editor by R. Papke

**DOI:** 10.3205/dgkh000394

**Published:** 2021-06-24

**Authors:** Martin Exner, Sanjay Bhattacharya, Jürgen Gebel, Peter Goroncy-Bermes, Philippe Hartemann, Peter Heeg, Carola Ilschner, Axel Kramer, Moi Lin Ling, Wolfgang Merkens, Peter Oltmanns, Frank Pitten, Manfred Rotter, Ricarda Maria Schmithausen, Hans-Günther Sonntag, Kathrin Steinhauer, Matthias Trautmann

**Affiliations:** 1 Institute of Hygiene and Public Health, Bonn University, Bonn, Germany; 2 Tata Medical Center, Kolkata, India; 3 Schülke & Mayr GmbH, Norderstedt, Germany; 4 Departement Environnement et Santé Publique S.E.R.E.S., Faculté de Médecine, Nancy, France; 5 Institute of Medical Microbiology and Hygiene, University of Tübingen, Germany; 6 Institute of Hygiene and Environmental Medicine, University Medicine Greifswald, Germany; 7 Infection Prevention & Control, Singapore General Hospital, Singapore; 8 IKI - Institut für Krankenhaushygiene & Infektionskontrolle GmbH, Gießen, Germany; 9 Hygiene Institute, Medical University Vienna, Austria; 10 Institute of Hygiene and Medical Microbiology, University of Heidelberg, Germany; 11 Department of Hospital Hygiene, Stuttgart Hospital, Stuttgart, Germany

## Authors’ statement

We are grateful for the question by Papke [1] because it addresses an important issue regarding the risk-conscious use of hand disinfectants. 

The KRINKO does not recommend adding quaternary ammonium compounds (QAC) to hand disinfectants without mentioning the active ingredient mecetronium ethylsulfate (MES). However, mecetronium ethylsulfate (syn. Ethylhexadecyldimethylammonium-ethylsulfate) is a surface-active aliphatic quaternary ammonium compound, so that this active ingredient is also included in the KRINKO’s disapproval recommendation.

MES was chosen as an example in our publication because it is used in hand disinfectants [2] as well as in shampoos [3], but the reasons against its wide use are little known to the user. In this respect, the question may help to clarify the situation. 

The use of MES in hand disinfectants should be avoided for the following reasons:

- In the results of 23 studies, no improvement in efficacy was achieved by adding 0.2% MES to a propanol-based hand disinfectant, tested according to EN 12791 [4]. The same applies to chlorhexidine digluconate and 2-phenylphenol [4]. Also for surgical hand disinfection, no improvement in efficacy was demonstrated by addition of MES [5].
- After dermal application under occlusive conditions for 24 h, the percutaneous absorption of MES in the rat was approx. 2% [6]. By extrapolation from dermal toxicity data, the absorption was calculated to be 3% [7].
- For the quaternary ammonium compounds benzalkonium chloride (BAC) and didecyldimethylammonium chloride (DDAC), blood levels of both compounds were measured for the first time in 2020 in a sample of healthy people, with quaternary ammonium compound (QAC) exposure (n=43) at concentrations with possible impact on cell metabolism in cell culture, reduction of mitochondrial function, and increase of inflammatory cytokines in volunteers in a dose-dependent manner [8]. The reason for the occurrence in humans is the wide use of both QAC in disinfectants and cleaning agents, for the preservation of care products and in food preparation. In surface disinfection, the main exposure to QAC probably occurs by inhalation (aerosol, dust) [9]. Unlike BAC and DDAC, MES is only used as an additive in hand disinfectants and shampoos. However, due to the dermal absorption of MES, a systemic toxic risk cannot be ruled out by daily application for hand disinfection up to >100 times, depending on the work area. Since the addition of MES to hand disinfectants is not associated with any improvement in efficacy, but absorptive toxic risks including unknown summation effects with BAC and DDAC may result, the use of hand disinfectants with the addition of MES should be discouraged in view of the new findings on the toxic hazard of BAC and DDAC, until the safety of the adsorptive uptake of MES has been proven by in-depth studies.
- A genetically encoded development of resistance with simultaneous increase in resistance to antibiotics has been demonstrated for BAC [10]. When passaged *in vitro* at subinhibitory concentrations, tolerance is induced by both BAC and DDAC [11], [12], [13], [14]. Although such studies have not been performed with MES, such a risk cannot be ruled out for MES due to the structural similarity, the mode of mechanism and, in particular, the application in a submicrobicidal concentration of 0.2%.

## Conclusions

The occurrence of QAC in the blood of healthy people is an alarm signal that exposure to antimicrobial active QAC must be limited to the indispensable minimum. Since the addition of MES to propanol-based hand disinfectants did not improve their efficacy, but the active ingredient is absorbed dermally, the use of hand disinfectants containing MES cannot be recommended until toxic long-term risks and the development of tolerance and resistance have been reliably ruled out.

## Notes

### Competing interests

The authors declare that they have no competing interests.
